# Overexpression of plasmalemmal vesicle-associated protein-1 in patient with cyanotic nephropathy: a case report

**DOI:** 10.1186/s12882-025-04046-x

**Published:** 2025-03-03

**Authors:** Yusuke Ushio, So Hirata, Shun Manabe, Mayuko Suyama, Ayano Tanaka, Momoko Seki, Haruka Kato, Kana Nomura, Anna Nakai, Hitoko Sumori, Yuki Kawaguchi, Shizuka Kobayashi, Shiho Makabe, Hiroshi Kataoka, Naoko Itoh, Sekiko Taneda, Kazuho Honda, Junichi Hoshino

**Affiliations:** 1 Department of Nephrology, Tokyo Women’S Medical University, 8-1, Kawada-Cho, Shinjuku-Ku, Tokyo, Japan; 2 Department of Surgical Pathology, Tokyo Women’S Medical University, 8-1, Kawada-Cho, Shinjuku-Ku, , Tokyo, Japan; 3 Department of Anatomy, Showa University School of Medicine, 1-5-8 Hatanodai, Shinagawa-ku, Tokyo, Japan

**Keywords:** Cyanotic nephropathy, PAL-E, Cyanotic congenital heart disease, Case report

## Abstract

**Background:**

Cyanotic nephropathy (CN) is a known complication of cyanotic congenital heart disease (CCHD). However, many aspects of its pathophysiology remain unclear.

**Case presentation:**

We report the case of a 29-year-old male with a history of tetralogy of Fallot. Renal biopsy revealed glomerular hypertrophy and focal segmental glomerulosclerosis. Electron microscopy revealed extensive endothelial cell damage. To investigate the etiology of endothelial cell damage, PAL-E staining was conducted, revealing staining along the glomerular capillary wall.

**Conclusion:**

This is the first report of PAL-E staining in CN, suggesting potential overexpression of PV-1. The association of PV-1 expression with endothelial cell damage indicates its role in the pathogenesis of CN.

## Introduction

Cyanotic nephropathy (CN) was first documented in 1953 by Meessen and Litton, who reported 28 autopsy cases of cyanotic congenital heart disease (CCHD) and observed a correlation between glomerular hypertrophy and the degree of cyanosis [1]. CN has long been recognized as a potential complication of CCHD, with rising concerns about the increasing number of patients developing nephropathy as the survival rate of CCHD patients improves [2].

The pathogenesis of CN is hypothesized to involve increased shear stress from polycythemia, which induces nitric oxide (NO) release and subsequent glomerular hypertrophy. However, despite glomerular hypertrophy being the primary pathological finding, a previous study found no association between glomerular size and CN development when comparing CN and non-CN patients [3]. Autopsies have revealed pathological findings such as glomerular hypertrophy, capillary dilatation, an increased number of glomerular capillaries, thickening or rupture of capillary walls, and mesangial expansion [4–6]. However, a comprehensive study of the pathophysiology of CN has yet to be conducted.

Pathologische Anatomie Leiden-Endothelium (PAL-E) staining colors veins and peritubular capillaries but does not typically stain glomerular endothelial cells. There have been reports of PAL-E staining observed in glomerular capillaries with endothelial cell damage [7], prompting investigation into the relationship between PAL-E staining and endothelial cell damage. In this report, we discuss a case of CN in which PAL-E staining was positive, along with its pathophysiology.

## Case presentation

A 29-year-old male with severe tetralogy of Fallot presented with 1 + microscopic hematuria, which had worsened over the past year. Concurrently, urinary testing revealed proteinuria escalating from 1 + to 3 + during the same period. We received a consultation from a pediatric cardiologist; and laboratory tests showed a creatinine (Cre) level of 0.77 mg/mL; eGFR of 98.2 mL/min/1.73m^2^; blood urea nitrogen (BUN) of 16.2 mg/dL; 1 + proteinuria; and microscopic hematuria (50–99/high power field (HPF)), suggestive of deteriorating kidney dysfunction. Cyanotic nephropathy complicated by IgA nephropathy was suspected, prompting his admission for a kidney biopsy.

Upon admission, laboratory findings were as follows: white blood cell count, 7740 /μL; red blood cell count, 7.74 × 10^6^ /μL; hemoglobin level, 21.3 g/dL; platelet count, 15.0 × 10^4^/μL; albumin level, 3.8 g/dL; Cre level, 0.78 mg/dL; eGFR, 96.9 mL/min/1.73m^2^; BUN, 11.5 mg/dL; sodium, 140 mEq/L; potassium, 4.5 mEq/L; chloride, 107 mEq/L; IgG, 1376 mg/dL; IgA, 485 mg/dL; IgM, 120 mg/dL; CH50, 42.3 U/mL; C3, 104.8 mg/dL; C4, 18.5 mg/dL; and vascular endothelial growth factor (VEGF), 941 pg/mL. Urinary tests showed a urinary protein/Cre ratio of 2.0 g/gCre and a urinary sediment red blood cell count of 50–99/HPF.

**Fig. 1:**
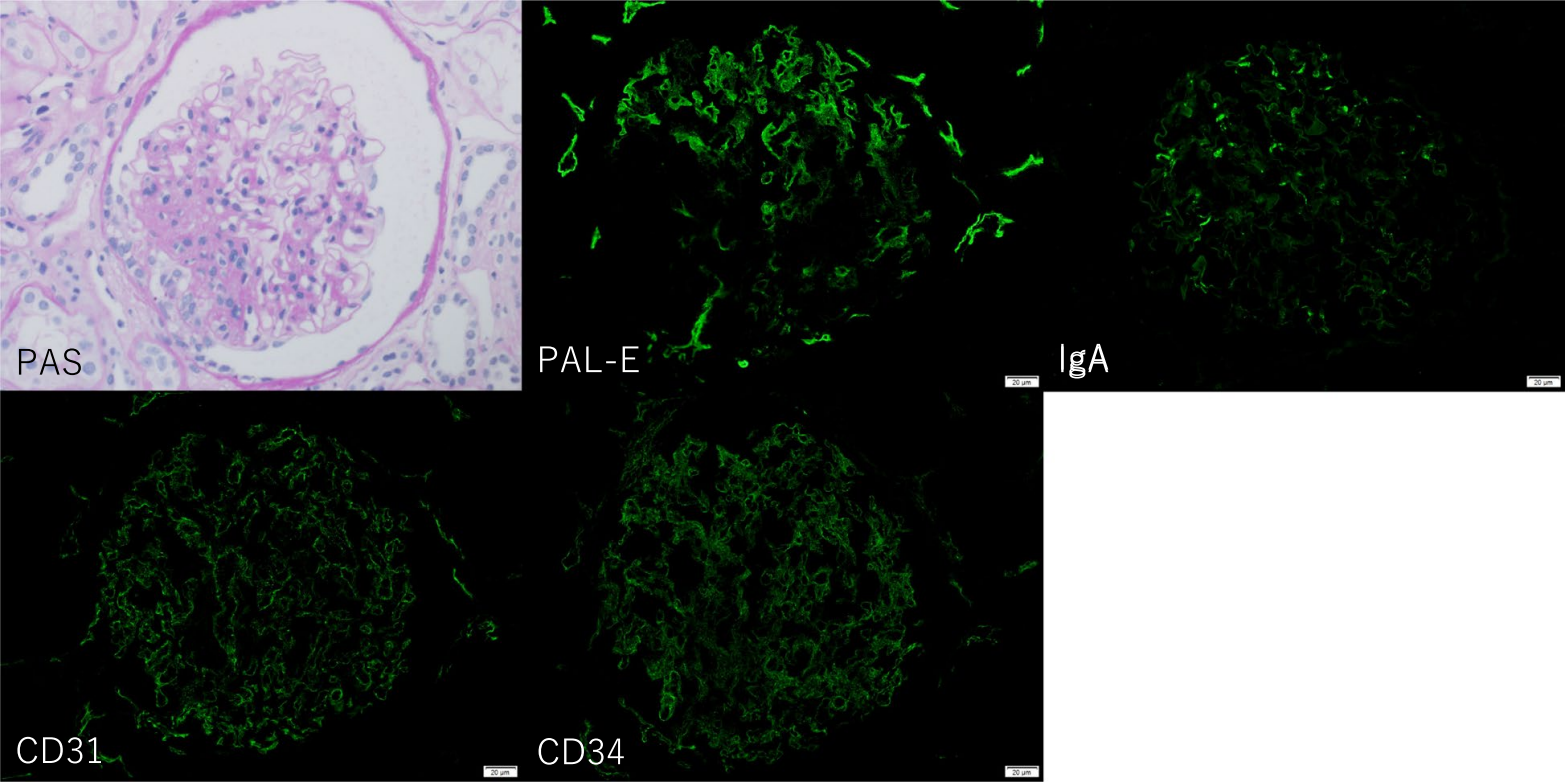
Kidney biopsy findings. Light microscopy showed segmental sclerotic perihilar lesion on PAS staining, × 200 original magnification. Immunofluorescence microscopy was used for IgA, CD31, CD34 and PAL-E, Original magnification × 400

**Fig. 2:**
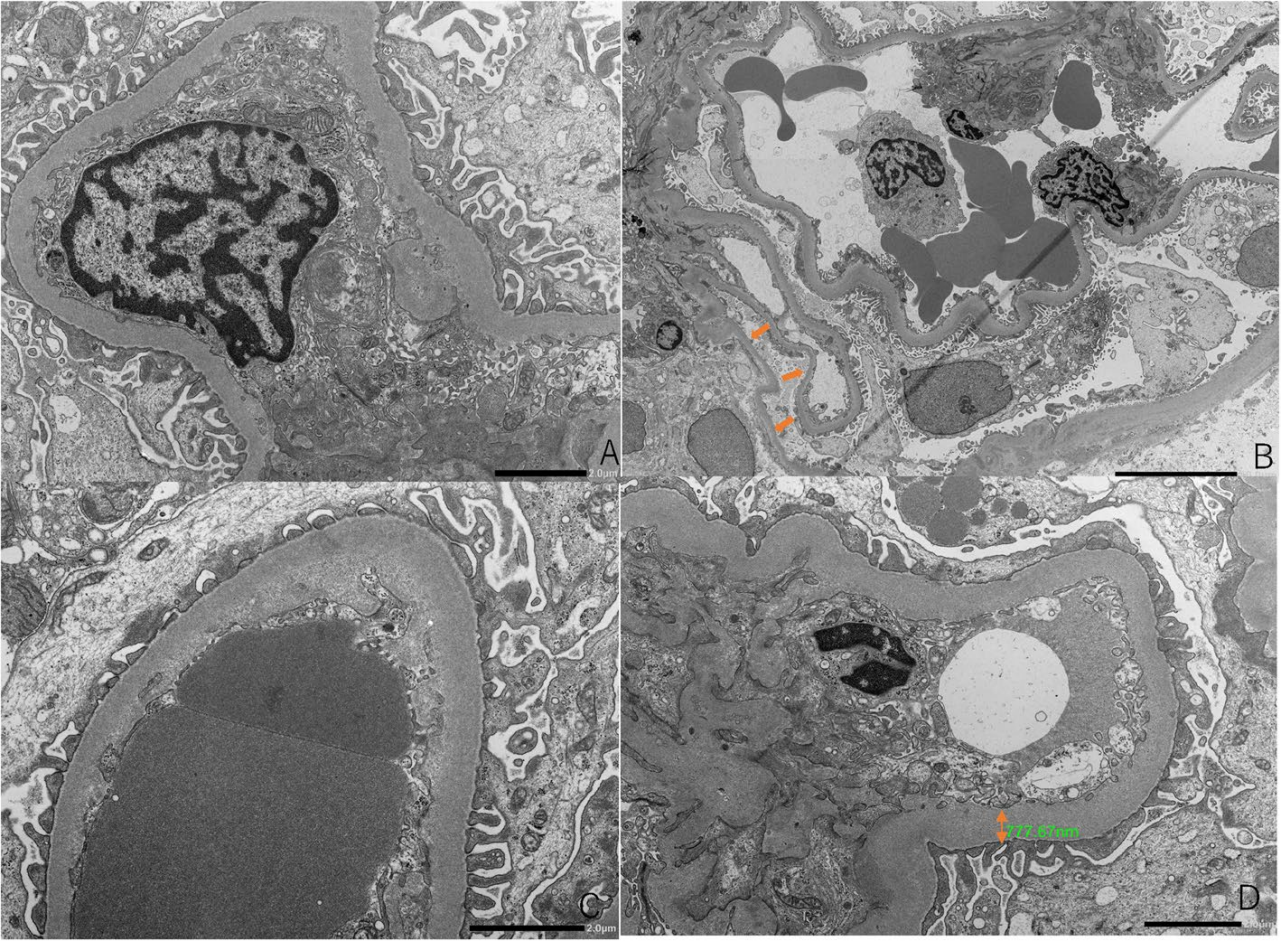
Electron microscopy (EM) showed endothelial cell swelling (original magnification × 4000 (**A**)). EM showed podocyte detachment(original magnification × 1000, (**B**)), subendothelial edema(original magnification × 4000 (**C**)), glomerular basement membrane was thick (777.67 nm, original magnification × 3000, (**D**))

A renal biopsy was conducted, and light microscopy revealed 42 glomeruli, 2 global sclerosis, and 1 focal segmental glomerulosclerosis (FSGS) lesion. The glomeruli displayed hypertrophy, with a mild increase in mesangial cells and matrix. The glomerulus with FSGS exhibited showed a perihilar variant. Immunofluorescence staining showed IgG( ±); IgA( +); IgM( ±); C1q(-); C3( ±); C4(-); CD31( +); CD34( +); and PAL-E( +) (Fig. 1). Electron microscopy showed endothelial cell swelling, subendothelial edema and glomerular basement membrane thickness (777.67 nm) (Fig. 2).

Based on these findings, a diagnosis of CN with endothelial damage was established. Treatment with an SGLT2 inhibitor in addition to an angiotensin-receptor blocker led to a decrease in urinary protein levels from 1.38 g/gCre to 0.28 g/gCre, with stable renal function.

## Discussion

CN is considered a complication of congenital cyanotic heart disease, and the number of cases that develop it increases with prolonged survival. Hongsawong et al. reported that hematocrit > 40%; platelets < 290,000; and waiting time before surgery are associated with CN [8]. Inatomi et al. [3] also compared patients with CCHD who developed CN with those who had not. Hematocrit values were higher in patients with CN than in those without CN, but no differences were found between these groups with respect to years of disease duration or oxygen saturation. These results support the previously reported hypothesis that increased shear stress induced by polycythemia vera is the main cause of CN; in the glomeruli of patients with CN, glomerular diameter was larger and the number of coagulation hooves was higher than in patients without CN. In the present case, the patient had risk factors relating to hematocrit and platelets, and his histopathology was consistent with CN, as the glomerular diameter was larger and the number of coagulations had increased. In addition, there were no metabolic abnormalities such as diabetes mellitus, but thickening of the basement membrane was observed. The cause of the thickening of the basement membrane has not been mentioned in CN in the past and is not clear, but non-immunological mechanisms may be involved, as there was no deposition of immune complexes.

PAL-E staining is known to primarily react with plasmalemmal vesicle-associated protein-1 (PV-1) expressed in endothelial cells of peritubular capillaries (PTC) and venous endothelial cells, but not with glomerular endothelial cells [8]. However, in transplanted kidneys with extensive glomerular endothelial cell damage, PV-1 was highly expressed in glomerular endothelial cells [7]. Furthermore, PV-1 is co-stained with caveolae. Diabetic nephropathy typically leads to endothelial cell damage, and upregulation of the Cav-1 expression [9, 10]. The aforementioned suggests that endothelial cell damage may be involved in the expression of PV-1. In the present case of CN developed secondary to congenital heart disease, we observed both glomerular hypertrophy and marked glomerular endothelial cell damage, and PAL-E staining, which is typically not seen in glomerular endothelial cells, in the glomerular capillaries. However, the mechanism behind endothelial cell damage in CN has not been previously discussed.

In CN, persistently high levels of VEGF in the bloodstream may contribute to endothelial cell damage. VEGF levels are elevated in diabetic nephropathy and FSGS, and may be associated with renal dysfunction [11]. VEGF stimulates endothelial cells to produce nitric oxide, which, when decreased in bioactivity, can lead to glomerular damage through endothelial cell proliferation and macrophage infiltration. Additionally, VEGF prompts the production of intercellular adhesion molecule-1 (ICAM-1), which may also contribute to endothelial cell damage.

ICAM-1 expression, induced by VEGF, increases vascular permeability by facilitating leukocyte adhesion to vascular endothelial cells [12–14]. In conditions like diabetic nephropathy, increased shear stress triggers ICAM-1 expression, leading to macrophage recruitment [15]. Similarly, reflux nephropathy, which involves shear stress, demonstrates upregulated ICAM-1 expression [16]. This suggests a potential association between ICAM-1 and endothelial cell damage in CN, where shear stress is also a contributing factor. However, the precise relationship remains unclear and necessitates further investigation.

**Figure3:**
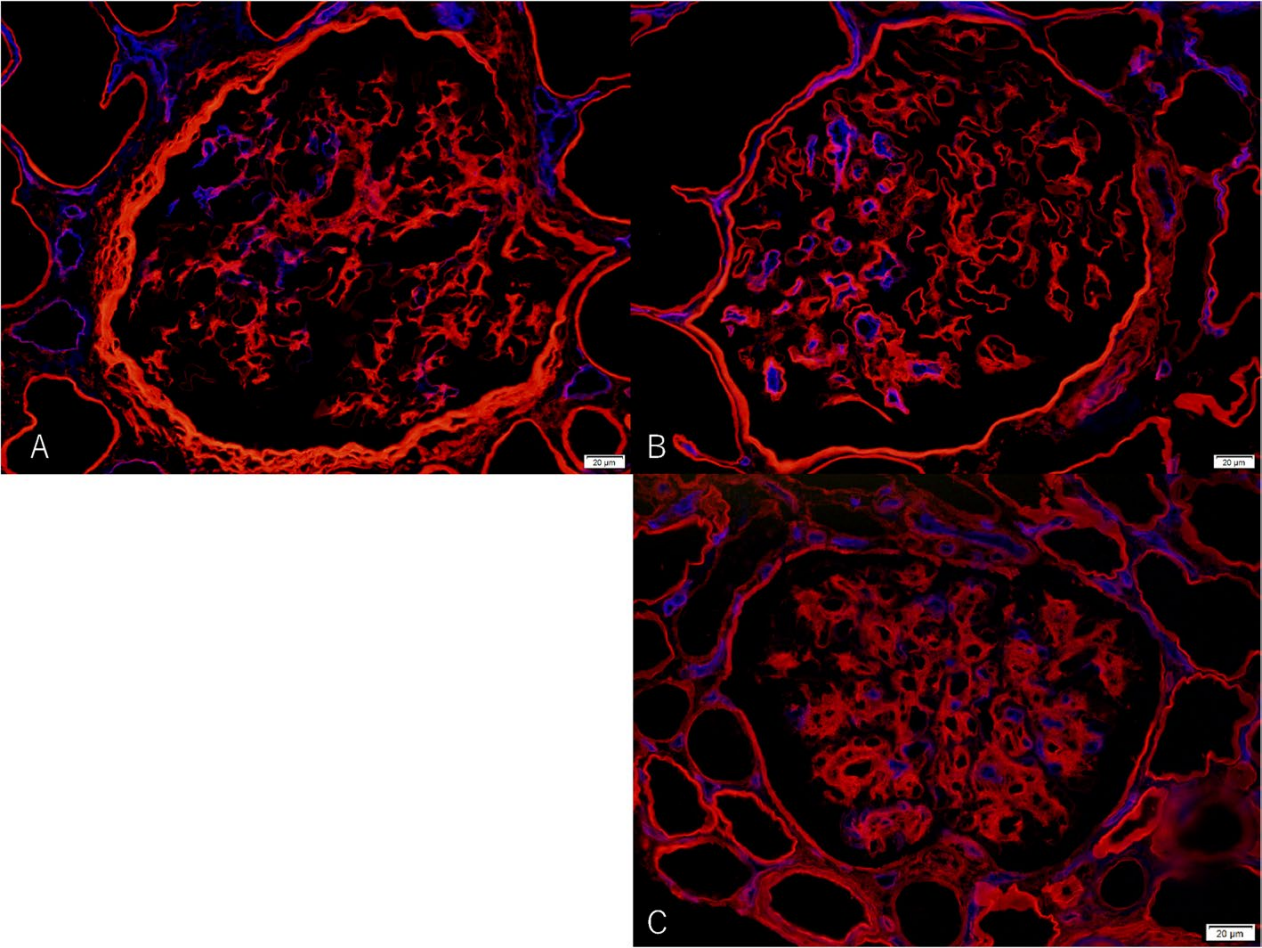
double immunofluorescence microscopy for PAL-E and alpha 2 subtype of collagen type IV (× 400, Blue: PAL-E, Red: alpha 2 subtype of type IV collagen, A. Cyanotic nephropathy

To further clarify the pathogenesis of endothelial cell damage, we performed double staining with alpha2 of collagen type IV and PAL-E to compare with diabetic nephropathy (DN) and transplant glomerulopathy (TGP), which are glomerular diseases causing endothelial cell damage (Fig. 3). DN and TGP showed staining in the glomerular endothelium as in the present case. While a mechanism of endothelial cell damage has been suggested for diabetic nephropathy as described above, Yamamoto et al. [7] reported that endothelial cell damage is associated with PAL-E in TGP. Though endothelial cell damage in TGP has been associated with immunological mechanisms, it is not clear whether CN is caused by immunological or non-immunological mechanisms. It is difficult to conclude whether CN causes endothelial cell damage by immunological or non-immunological mechanisms. In addition, more capillary lumens were stained with PAL-E staining in TGP than in CN and DN in double staining. This may be related to the difference in staining, as CN and DN are associated with shear stress, whereas TGP is associated with immunological mechanisms such as antibodies. However, it was difficult to clarify why segmental endothelial cell damage occurred in this report.

## Conclusion

We encountered a case in which PAL-E indicated potential overexpression of PV-1 in CN, suggesting a link to endothelial cell damage. Despite this observation, many aspects of the pathophysiology of CN remain unclear. Further accumulation of case studies are required for a more comprehensive understanding of its pathogenesis.

## Data Availability

No datasets were generated or analysed during the current study.
